# Pathogenesis

**Published:** 1869-04-01

**Authors:** Jules Lemaire


### CHICAGO MEDICAL JOURNAL.

Vol. XXVI.—APRIL 1, 1869.—No. 7.

### TRANSLATIONS.

### *Pathogenesis.*

*Are Typhus, Cholera, Plague, Yellow Fever, Dysentery, Intermittent Fevers and Hospital Gangrene due to Infusoria which perform the functions of ferments? Memoir read to the Academy of Sciences (Paris). By Doctor Jules Lemaire.*

The history of these diseases ascends to the very cradle of Medicine. The numerous descriptions of them which have been transmitted to us, have taught us, I may even say, to render homage to the authors who have devoted themselves to their investigation, since they have described admirably the conditions under which these diseases originate, and all that clinical observation and pathological anatomy can teach.

But they left for solution two important points of their history, of which one is the consequence of the other. I refer to the nature of these diseases, and the rational treatment which should be applied to them.

In fact there can be discovered upon this subject, amongst authors, only a series of hypotheses, accepted to-day by some, rejected to-morrow by others. A few physicians, recognizing the insufficiency of these theories, have preferred to acknowledge that the nature of these diseases was unknown to them. Finally, there are some who, like M. Roche, of the Academy of Medicine, have written: "The essence, or the intimate nature of diseases, is unknown to us. Object of useless researches, subject of ridiculous conjectures in all times, it escapes us, and will ever escape us. It would be idle to seek further to discover it. It is ever impenetrable." (*Lettres sur le cholèra.*)

This uncertainty appeared to me to be the cause of the diverse and unsatisfactory modes of treatment which have been recommended to combat them. Such was the state of science upon this point in 1860. Since this epoch I have published numerous investigations into the nature of ferments, of virus, of poisons, and of miasmata, which have for their principal aim the elucidation of this important point of general pathology, in order to establish the therapeutics of these diseases upon more certain bases. I have even condensed into the form of a proposition since 1862 (See Moniteur Scientifique du Docteur Quesneville, Octobre) my opinion upon this important question. (See my book upon Phenic Acid.)

To-day I shall have the honor to submit to the appreciation of the Academy the results of new investigations, which, associated with those that I have already communicated, appear to me to demonstrate that these diseases are the work of infusoria which play the part of ferments.

The title of my new work appears to me to indicate clearly my object. But the solution of this question is not so simple as it appears. It appears to me that the problem should be thus stated: *These diseases being given, to demonstrate in the media in which they originate, and in the organism, the existence of infusoria which play the part of ferments.*

To one who studies the subject with care, other embarrassing questions speedily present themselves, whose solution it is nevertheless important to give. These questions are the following:

1st. If these minute organisms originate these diseases, how is it that man and other animals have not disappeared from the face of the globe long since; since it has been demonstrated that these infusoria exist everywhere, in the air, in the water, in the earth, in our food, upon the body of man in health or disease, upon vegetables, etc., etc.

2d. Why, if they are due to microphytes and microzoa, do they disappear, since these little beings reproduce themselves and multiply in incalculable proportions. Does it not seem that their work would only terminate after the destruction of the human species and of other animals?

3d. Why does it happen in an endemic or epidemic, that many individuals are not attacked?

4th. Can the alteratives of the solids and liquids which are observed in these diseases be attributed to them?

5th. Are the phenomena thus presented due to microphytes or microzoa?

6th. Is each one of these diseases due to a special microphyte or microzon, or are they indeed the result of the action of several?

It must not be misunderstood that these constitute a portion of the problem. In spite of the difficulties which they present, I shall treat them successively in the course of this work. I shall commence by making an inventory of the facts already acquired in the history of these diseases. By classifying and criticising them, and by adding thereto my own observations I hope to throw the clearest light upon this question.

I shall first discuss a point which seems to me important, viz.: Do these diseases form distinct species?

The appellations which have been assigned to them are based upon their dominant symptoms. They appear to me to have no scientific value. If it is attempted to solve the problem which I have just proposed, I think it will be necessary to take into consideration these different appellations, because, in describing these diseases as of distinct species, it seems that science has been rather embarrassed than aided. Proposing to remove the subject from the abstract to the concrete, as my illustrious master, M. Chevreul would say, I cannot accept, as characteristic of these diseases, names based upon a symptom, because this symptom may, perhaps, be produced by very different causes, Moreover, they may all be found associated in a single one of these diseases. All, as we shall see, originate from a common cause, and have common characteristics. This is my reason for insisting upon this point.

It is sufficient to consult the nomenclature of these diseases to conclude that a large number of authors have not accepted the designations which we are now considering.

In the midst of this confusion of names, and of opinions which have originated them, there will be found landmarks planted by the princes of science, which seem to me, to indicate the path leading to the demonstration of the unity of species of these diseases.

Hippocrates, whose teachings have been religiously followed up to the end of the last century, does not separate the plague from pestilential fevers. Under these denominations a great number of authors have described the fever of prisons, of ambulances, of ships, of camps (described at our epoch under the name of typhus and of typhoid fevers). Cholera has been, likewise, described under the name plague.

Galen, perceiving that in these diseases the blood becomes putrid, designated them under the name, putrid fever. The opinions of Galen reigned supreme up to the end of the last century, an epoch at which science, like empires, experienced a violent shock.

The plague of Egypt is described by Hoffmann, Swèdiaur, Vogel and others, under the name typhus.

Yellow fever is called by Sauvages typhus icterodes; and typhus miasmatic, ataxic, putrid and yellow by M. Bailly. Others have called it also putrid or malignant fever, like that of prisons, ambulances, etc., already referred to. Cholera has been termed by several authors, and very recently again by M. Pellarin, Indian typhus.

A great number of physicians since Galen admit a choleraic intermittent fever, and cholera has been considered as a pernicious intermittent fever.

Cullen, whose authority is so great, says that yellow fever, intermittent, malignant and putrid, bilious, mesenteric and catarrhal fevers should not be separated from typhus, of which they are merely varieties.

We find in this rapid analysis an important indication: it is the tendency of eminent men towards unity of species of these diseases.

If we interrogate history still further upon this subject, we find facts of still greater importance.

Since the time of Hippocrates, physicians and veterinarians have observed that wherever matter in a state of putrefactive fermentation exists in great abundance, or wherever there may be large collections of men or of animals in health, either within restricted limits, or in the open air as in camps, serious diseases, transmissible, according to some, non-transmisible, according to others, originate.

*For Example:* Cholera is endemic upon the borders of the Ganges, which constitutes the place of sepulture of the Hindus.

Yellow fever originates upon the borders of marshes, principally at the mouths of rivers where putrescent matters are massed in considerable quantity. Plague is endemic in Egypt, which is covered with marshes. It is especially upon that portion of the population which lives in profound misery, in common with domestic animals, in habitations where filth of all sorts accumulates, that this disease rages.

Intermittent fever, dysentery, the cachexia or rot of sheep, which rages also amongst bovine cattle, originate wherever stagnant water containing putrescent matter exists.

Accidental foci of putrid fermentation, constituted of accumulations of dead bodies of men or of animals, as, after great battles or epizootics, have likewise originated in every age, grave maladies, which have been described under the name of plague, pestilential fever, malignant, putrid or typhus fever, and of hospital gangrene.

My experiments upon the nature of miasmata furnished by the human body in health, which engender the diseases already referred to, demonstrate that they originate also from matters in the state of putrid fermentation.

It cannot be ignored that there is for all these diseases a common origin: the emanations from matters in the state of putrid fermentation. If I now analyze and compare the lesions and the symptoms which are observed in these diseases, it will be easy for me to prove that this common cause produces common effects; for example: the buboes, the carbuncles, the petechiæ, the gangrene, the gastro-intestinal symptoms, and others which are observed in individuals subjects of plague, exist in hospital typhus, in severe typhoid fever, in yellow fever and in the dysentery of hot climates. This dysentery often follows intermittent fever, or even complicates it, or is terminated by it.

Hippocrates, Torti, Willis, Morton, Monget, Sauvages, Morgagni, and physicians of our epoch admit a choleraic intermittent fever, and as has been already stated, cholera has been considered a pernicious intermittent fever.

In cholera, after the first symptoms, a very decided typhoid condition has been frequently determined.

The intermittent fevers of Africa are frequently initiated by a general chill of the body, which persists until death; * under the name of algid fever. These fevers are accompanied by vomiting, diarrhœa, epigastric uneasiness, dyspnea as in cholera. In yellow fever the urinary secretion is suppressed as in cholera.

---

* This frequently occurs in the intermittents of the extreme Southern States of this country. *Teste meipsuro.* TRANSLATOR.

Excellent physicians have collected numerous observations to demonstrate that intermittent fever may be transmitted from one individual to another.

I might cite still other common characteristics which are observed in all these diseases. I have selected the most important in order to fix the attention more firmly.

This comparison appeared to me of the highest importance, because it demonstrates, in the diseases described as of distinct species, the existence of a great number of common symptoms, of identical lesions or alterations.

Physicians who have studied with care the history of diseases know that certain symptoms may induce variations in the morbid species, but cannot change it.

All these facts borrowed from the history of these diseases inflict a heavy blow upon the edifice of the past. They seem to me to indicate the necessity for a reformation of the nomenclature of these diseases. I shall produce others which seem to me to establish their identity in nature, and which permit me to hope that soon they will be ranged in the great class of parasitic diseases.

Numerous cases of poisoning have been occasioned by putrescent food. Lesions and symptoms analogous to those which occur in the diseases now under consideration, have been observed. Physicians know that the internal use of marsh water produces the same effects of acute or slow intoxication, as the absorption of their emanations by the respiratory surface.

According to M. Roche, of the Academy of Medicine, intermittent fever has been produced by introducing into animals the vapor of water condensed over marshes.

Numerous experiments have been made upon animals with putrescent matters. Some have been caused to inspire their emanations; in others they have been introduced into the stomach or into the rectum, or by subcutaneous inoculation, or injected into the veins. In these different experiments the results have been the same. Grave symptoms or death have been the consequence. Thus, when men or animals introduce into their organisms, by the modes just mentioned, putrescent matters, the same effects are observed.

The experiments which I published long ago, explain, and seem to me to demonstrate the cause of the identity of these results.

In fact, I have proved that the gases and vapors which are disengaged from all putrescent matters carry with them in considerable quantities the reproductive bodies of microphytes and of microzoa which immediately provoke fermentation in fresh animal or vegetable substances. Now the tenuity of these bodies permits them, not only to remain in suspension in the atmosphere, but even to penetrate into the organism through the respiratory ducts. By the inoculation, by the injection, or by the introduction of these matters into the digestive tube, both the reproductive bodies and infusoria in the adult state are caused to penetrate.

If these little beings occasion these diseases, they ought to be discovered in the organisms of the individuals attacked by them. This fact is now incontestable. Bacteria and vibriones exist in the circulating blood of typhic and variolous subjects; in the disease termed ———————— in anthrax, in moist gangrene, and in malignant pustule. These same animalculi, as also monads and cerco-monads, exist, likewise, in the dejections of typhic, choleraic and dysenteric subjects. Many observers, as well as I, myself, have determined this. The discovery of these infusoria in all these pathological cases, appears to me to possess immense importance, since, in the normal state they do not exist in the organism, as will be seen hereafter.

I have made experiments upon myself in a state of health, with a view to ascertain if an exclusively animal or vegetable regimen favored or opposed their development in the residua of digestion. These experiments which gave me negative results, have assumed subsequently for me real importance.

Having been violently attacked, some months later, by cholera, I made of it a new study, which enabled me to compare the results. Eight days after the commencement of the attack I could scarcely keep myself upright; I examined not only the fæces, but also all the products of the secretion and of respiration. Neither the watery vapor of respiration collected with the precautions I have recommended, nor the nasal mucus, nor the urine, contained infusoria. But I discovered in the fæces myriads of bacteria termo, and vibriones, liniear, angular and catenary.

Some of these last have seven rings. I also found spirilli volutantes, little monads and circomonata crassicaudæ. This observation compared with the first appeared to me of the highest importance, and what gave it still more significance, was the fact that two months after my recovery, examining anew these matters with the microscope, I no longer found infusoria. Their presence, therefore, was certainly due to cholera. Having perspired freely, I recognized in the matters collected upon the different portions of she skin, spores analogous to those which I described in my memoir upon the nature of miasmata, and a considerable quantity of bacteria and little vibriones. Having been compelled to neglect during eight days the care of my mouth, I found in abundance bacteria, linear vibriones, spirilli and monads. Finally, removing a flannel waistcoat which I had worn four days, I caused it, still moist and warm to be washed in a small quantity of distilled water. I immediately examined this liquid with the microscope. I discovered in it, in abundance, the same species of infusoria as those whose existence upon the skin I had already determined.

The experiment, which demonstrates the presence of infusoria in the clothing, explains how these, and also bedclothing, have so often propagated these diseases. This is not all: blood collected during life from man and from animals suffering from typhus or variola, and containing bacteria and vibriones has been inoculated, or injected into the veins of dogs, of sheep and of rabbits in perfect health.

The bacteria and the vibriones would reproduce and multiply themselves, determining formidable symptoms, and almost always death. MM. Cose, and Feltz, who have experimented, have succeeded in reproducing in rabbits ten successive generations of bacteria, as M. Virchow has done with trichinæ. They ascertained that the more these generations are repeated the more active they become, which is demonstrated by the more prompt rapidity of the accidents which they occasion. I published some experiments which established that bacteria desiccated return rapidly to life when they are bathed in water at 25° centigrade. MM. Cose and Feltz have established the same fact upon blood containing these animalculæ which they had desiccated. This blood inoculated, in the dry state, into rabbits, did not induce death, whilst, when previously moistened and inoculated in the same proportions in other rabbits, death was the speedy consequence. I shall hereafter refer to this fact.

What gives great importance to the experiments of the learned professors of the Faculty of Medicine, Strasbourg, is the fact that they corrected them by comparative experiments made with normal blood. These last demonstrate that a considerable quantity of blood taken from a healthy man may be injected under the skin of rabbits without inducing augmentation of temperature, nor disturbance.

Before concluding this first treatise, I will recall the discussion which I established—formerly in order to prove that the causes of transmissible maladies could not be attributed to chemical agents nor to poisons, nor to dead matters; because neither can reproduce and multiply itself, whilst the essential characteristic of the diseases which we are considering is to reproduce and multiply itself in proportions sometimes considerable. Now if, as I have done long since, one should kill these infusoria either with coal tar or its compounds, or with other substances, not only is the fermentation arrested, but at the same time moreover, these matters already profoundly altered, are deprived of their power to induce it elsewhere, either by their exhala-

:ions, by contact, or by inoculations. From being dangerous as they were, they become, after the death of the infusoria, entirely inoffensive. Making applications of these facts to the therapeutics of authrax, of moist gangrene of malignant pustule (charbon) of typhus fever, of dysentery, of cholera, of tinea, of the itch, of dissecting wounds (of which last one was received in opening a horse dead of glanders), remarkable results have been obtained, (*V de l'acide phinique.*) All the facts which I have just related in this sketch are linked together. They appeared to me of the highest importance for the solution of this question. With these little beings, all the most difficult points which the history of these diseases presents can be scientifically resolved, as I hope to demonstrate in the sequence to this work.

